# Enhanced bacterial cancer therapy delivering therapeutic RNA interference of *c-Myc*

**DOI:** 10.1186/s13578-024-01206-8

**Published:** 2024-03-23

**Authors:** Jason S. Williams, Adam T. Higgins, Katie J. Stott, Carly Thomas, Lydia Farrell, Cleo S. Bonnet, Severina Peneva, Anna V. Derrick, Trevor Hay, Tianqi Wang, Claire Morgan, Sarah Dwyer, Joshua D’Ambrogio, Catherine Hogan, Matthew J. Smalley, Lee Parry, Paul Dyson

**Affiliations:** 1 Institute of Life Science, School of Medicine, Swansea University, Singleton Park, Swansea, SA2 8PP UK; 2 European Cancer Stem Cell Research Institute, School of Biosciences, Cardiff University, Hadyn Ellis Building, Maindy Road, Cathays, Cardiff, CF24 4HQ UK

**Keywords:** Bacterial therapy, RNAi, Colorectal cancer & breast cancer

## Abstract

**Background:**

Bacterial cancer therapy was first trialled in patients at the end of the nineteenth century. More recently, tumour-targeting bacteria have been harnessed to deliver plasmid-expressed therapeutic interfering RNA to a range of solid tumours. A major limitation to clinical translation of this is the short-term nature of RNA interference in vivo due to plasmid instability. To overcome this, we sought to develop tumour-targeting attenuated bacteria that stably express shRNA by virtue of integration of an expression cassette within the bacterial chromosome and demonstrate therapeutic efficacy in vitro and in vivo.

**Results:**

The attenuated tumour targeting *Salmonella typhimurium* SL7207 strain was modified to carry chromosomally integrated shRNA expression cassettes at the *xylA* locus. The colorectal cancer cell lines SW480, HCT116 and breast cancer cell line MCF7 were used to demonstrate the ability of these modified strains to perform intracellular infection and deliver effective RNA and protein knockdown of the target gene *c-Myc*. In vivo therapeutic efficacy was demonstrated using the *Lgr5creER*^*T2*^*Apc*^*flx/flx*^ and *BlgCreBrca2*^*flx/fl*^*p53*^*flx/flx*^ orthotopic immunocompetent mouse models of colorectal and breast cancer, respectively. In vitro co-cultures of breast and colorectal cancer cell lines with modified SL7207 demonstrated a significant 50–95% (*P* < 0.01) reduction in RNA and protein expression with *SL7207/c-Myc* targeted strains. In vivo, following establishment of tumour tissue, a single intra-peritoneal administration of 1 × 10^6^ CFU of *SL7207/c-Myc* was sufficient to permit tumour colonisation and significantly extend survival with no overt toxicity in control animals.

**Conclusions:**

In summary we have demonstrated that tumour tropic bacteria can be modified to safely deliver therapeutic levels of gene knockdown. This technology has the potential to specifically target primary and secondary solid tumours with personalised therapeutic payloads, providing new multi-cancer detection and treatment options with minimal off-target effects. Further understanding of the tropism mechanisms and impact on host immunity and microbiome is required to progress to clinical translation.

**Supplementary Information:**

The online version contains supplementary material available at 10.1186/s13578-024-01206-8.

## Introduction

RNA interference (RNAi) suppresses target gene expression and is consequently a rational therapeutic strategy for disease conditions caused by genetic mutations or by aberrant expression of a gene. While some small-molecule inhibitors and monoclonal antibodies have proved successful as cancer treatments [1, 2], these therapeutic strategies cannot be used to inhibit many identified important cancer therapy targets. For example, c-Myc is a major driver of tumorigenesis that impacts on cell growth, the cell cycle, metabolism, and cell survival. *c-Myc* overexpression in tumour cells results from a variety of mutations with consequent cell proliferation [3]. This deregulation of *c-Myc* is observed in approximately 70% of human cancers [4], and studies using transgenic mouse models to control *c-Myc* expression indicate that *c-Myc* inhibition can result in tumour regression [5, 6]. Consequently, *c-Myc* is an attractive target for cancer therapy. However, the architecture of this transcription factor does not present good opportunities for small-molecule interactions that can inhibit its function. Moreover, as it is localized to the nucleus it is inaccessible to antibody-based therapies. For these reasons, despite having a critical role in tumorigenesis, c-Myc is widely considered ‘undruggable’.

Consequently, RNAi of *c-Myc* is an attractive therapeutic prospect. However, to date the most significant hurdle to the successful use of small RNA therapeutics is their delivery to diseased tissue. Unprotected small interfering RNA (siRNA) is unstable in the blood stream due to the activities of serum nucleases and rapid renal clearance, leading to degradation and a short half-life [7]. Some progress has been made to chemically modify siRNA, providing greater serum stability for systemic RNAi [8], but these advances do not address specific delivery to diseased tissue. The development of various nanoparticulate systems to package and deliver siRNA have been described [9, 10], and by functionalising these systems it is possible to achieve some tissue tropism, particularly as therapeutics for hepatic diseases, including tumours of the liver.

Bacterial cancer therapy has a long history. In 1813, French physician Arsène-Hippolyte Vautier reported tumour regression in patients with severe infections from *Clostridium perfringens* [11]. In 1867, German physician Wilhelm Busch observed cancer remission when a patient contracted erysipelas, now known as *Streptococcus pyogenes* [12]. Clinical applications followed, pioneered by William B. Coley in New York, using combinations of killed *S. pyogenes* and *Serratia marcescens* [13, 14], giving survival rates among treated patients similar to current rates for those receiving chemotherapy [15]. Despite this, it is only relatively recently that interest in this therapeutic approach has been rekindled. Contemporary approaches employ attenuated versions of live pathogenic bacteria [16, 17] that exhibit satisfactory safety profiles in both healthy and tumour-bearing animals [18, 19]. Following their administration, these attenuated bacteria are cleared in hours to several days from the circulation, liver, and spleen [18, 20]. Bacterial tumour tropism is poorly understood, but tumour-specific features of immune suppression and deregulated metabolism likely provide the bacteria both immune-protection and a nutrient source [21–23]. Although these tumour-colonising bacteria may promote cell death by, for example, competing for nutrients, the principle therapeutic effect is believed to be due to subsequent stimulation of the immune system that then targets tumour cells infected with the bacteria [24–27].

Attenuated non-virulent mutants of the facultative anaerobe *Salmonella enterica* serovar Typhimurium (*S. typhimurium*) exhibit high tumour-tropism and can replicate in both hypoxic and normoxic tumour regions in vivo, with evidence from experimental models indicating that they are the most efficient anti-tumour bacteria tested thus far [12, 28–31]. *S. typhimurium* is a cell-invasive bacterium; the major determinant of this invasiveness is a Type 3 Secretion System, a molecular syringe that injects effector proteins directly into target host cells. These effector molecules cooperatively manipulate host cell signalling pathways that ultimately result in internalization of the bacteria, although the bacterium has evolved multiple seemingly redundant mechanisms to invade a range of host cell types [32]. Consequently, it has been possible to harness this bacterium to deliver a range of potential therapeutic payloads to tumour cells [33]. This includes two different strategies to deliver RNA interference: either by manipulating the bacteria to deliver a mammalian expression vector that encodes short-hairpin RNA (shRNA) [34] or by plasmid-driven expression of shRNA in the bacteria themselves [35]. The latter approach has also been demonstrated using *Escherichia coli* [36]. Both approaches are reliant on tumour cell colonisation by the bacteria and subsequent release of either DNA or RNA into the tumour cell cytoplasm. However, the longevity of target gene RNAi is compromised by the instability of either the mammalian vector or the bacterial plasmid in the absence of a selection pressure (e.g. antibiotic resistance) in vivo. Consequently, although transient RNAi is observed both in vitro and in experimental animal models, neither approach has led to clinical applications. Here we describe the derivation of tumour-targeting attenuated *S. typhimurium* engineered to provide prolonged therapeutic RNAi of the oncogenic driver *c-Myc* in immunocompetent animal models of colorectal and breast cancer.

## Results

### Derivation and stability of recombinant ***S. typhimurium*** SL7207

**Fig. 1:**
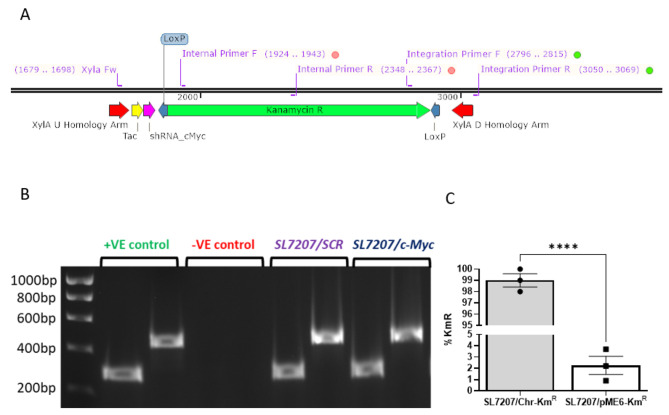
Design and validation of SL7207 synthetic expression cassette strains. (**A**) Schematic illustrating components and location of validation primers for incorporation of a synthetic shRNA expression cassette into the xylA locus of Salmonella strain SL7207. Two primer sets were used to test that the cassette was integrated (red dot internal primer set) and was in the correct orientation (green dot integration primer set). (**B**) The expected PCR products of 274 bp (integration primers) and 444 bp (internal primers) for both the positive control and the scrambled (SCR) and c-Myc shRNA strains were observed. (**C**) Percentage of KmR cells five days (∼ 80 divisions) following kanamycin withdrawal demonstrating the retention of chromosomal (SL7207/Chr-KmR) versus vector (SL7207/pME6-KmR) derived resistance (**** *P* < 0.0001; unpaired T-test)

We designed synthetic expression cassettes incorporating a strong constitutive *tac* promoter driving expression of either shRNA targeting c*-Myc* (*SL7207/c-Myc*) or a scrambled shRNA (*SL7207/SCR*), adjacent to a kanamycin resistance gene flanked by *loxP* sites, the latter to allow deletion of the antibiotic resistance once the cassettes had been integrated in the *S. typhimurium* SL7207 chromosome (Fig. 1A). In addition, the synthetic cassettes incorporated flanking sequences of 80 bp homologous to the *S. typhimurium xylA* gene to enable their integration by lambda Red recombination [37] at this chromosomal locus. We reasoned that the *xylA* gene, encoding xylose isomerase, would be inessential to the bacteria when colonising a solid tumour and hence could be disrupted by integration of the cassettes. Integration and the correct orientation of the recombinant kanamycin resistant (Km^R^) *S. typhimurium* SL7207 cassette was validated by colony PCR using both internal cassette and integration-specific primer pairs (Fig. 1B & Supplementary Table 1). With complete sequencing using Oxford Nanopore technology of targeted strains confirming integration of the full cassette at the target site with no off-target recombination (data not shown).

The stability of the cassettes was determined by passaging recombinant strains in the absence of antibiotic selection over an extended period and assessing the retention of the kanamycin resistance gene (*SL7207/Chr-Km*^*R*^). For comparison, a *S. typhimurium SL7207* strain containing a cloning vector encoding kanamycin resistance was sub-cultured in parallel (*SL7207/pME6-Km*^*R*^) [38]. Triplicate cultures of the two strains were grown in LB medium without antibiotic selection (to mimic growth conditions *in cellulo/in vivo*) for 5 days, subculturing every 12 h. Kanamycin resistant cells were quantified as a proportion of total cell number on each day of subculture (by plating serial dilutions on LB and LB + Km plates and subsequently enumerating colony numbers). After 5 days, 98–100% (+/- 1%) of cells of the chromosomally integrated strain retained kanamycin resistance, significantly more than the 2.3% (+/- 1.4%) of vector integrated strain (*P* > 0.0001; Fig. 1C).

### Bacterial-mediated RNAi of ***c-Myc*** in cultured cancer cell lines

**Fig. 2:**
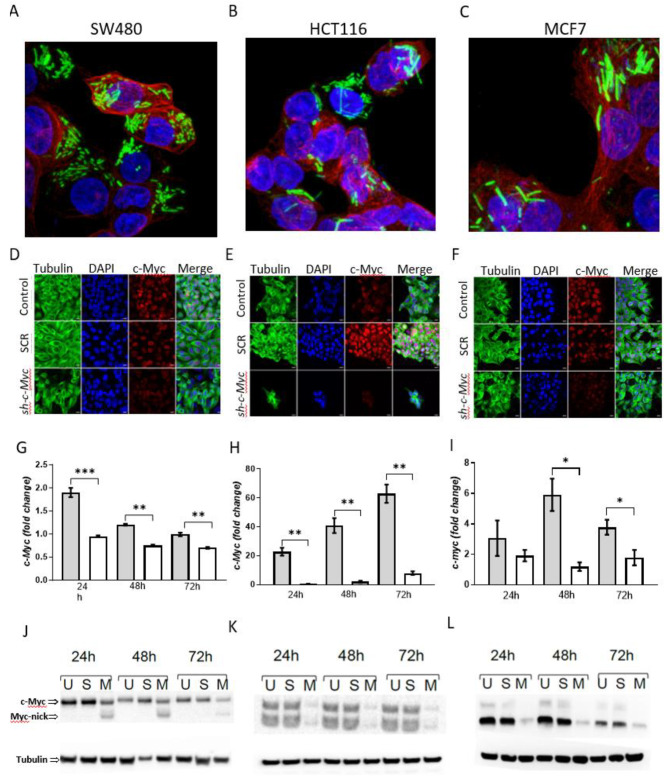
Bacterial-mediated RNAi of *c-Myc* in colorectal and breast cancer cell lines. (**A**-**C**) Immunofluorescence staining demonstrating intracellular infection of cell lines SW480, HCT116 and MCF7 (Red-B-actin; Blue-DAPI & green-bacteria). (**D**-**F**) Immunofluorescence staining demonstrating nuclear specific reduction of c-Myc protein at 24 h, 48 and 72 h following infection with *SL7207/c-Myc* strains in comparison to uninfected control and *SL7207/SCR* (D-SW480, E- HCT116 & F-MCF7). (**G**-**I**) qRT-PCR analysis of *c-Myc* expression at indicated time-points after infection with *SL7207-SCR* (shaded bars) and *SL7207-c-Myc* (white bars)(G-SW480, H-HCT116 & I-MCF7). Significant reduction of *c-Myc* mRNA was observed at timepoints indicated, with *c-Myc* mRNA levels normalised to *Gapdh, Hsp909a* and *ActB* mRNAs (t-test pairwise comparisons with * = *P* < 0.01, ** = *P* < 0.001 and *** = *P* < 0.0001). (**J**-**L**) Western analysis of protein samples from untreated cells (U), cells infected with SL7207/scr (S), and SL7207/*c-Myc (M)* demonstrating a specific semi-quantitative reduction in the abundance of c-Myc (J-SW480, K-HCT116 & L-MCF7)

The ability of *SL7207/c-Myc* to knockdown gene expression in vitro was assessed using confluent cultures of colorectal cancer (CRC) cell lines HCT116 and SW480 plus breast cancer line MCF7. Cells were infected at a multiplicity of infection of 1:1000 and RNAi of *c-Myc* assessed by immunofluorescence, qRT-PCR and Western blotting (Fig. 2). At 72 h we confirmed intracellular infection (Fig. 2A-C), using an *SL7207 s*train tagged with GFP, and visible reduction of c-Myc levels in all three cell lines (Fig. 2D-F). A significant reduction in the abundance of *c-Myc* mRNA was confirmed at 3 time points (24 h, 48 and 72 h) post-infection due to bacterial delivery of *SL7207*/*c-Myc* compared to a *SL7207/SCR* (Fig. 2G-I). RNAi was accompanied by a corresponding reduction of the protein, although with notable differences in the forms of c-Myc expressed in the different cell lines (Fig. 2J-L). Two major forms of the c-Myc protein have previously been detected in both HCT116 and SW480 colorectal cells lines: the complete 439 amino acid c-Myc protein and a C-terminal truncated 298 amino acid c-Myc-nick protein, derived from the former by calpain-dependent proteolytic cleavage [39]. In SW480 cells, the appearance of detectable c-Myc-nick correlated with RNAi of c-Myc, possibly as a cellular stress response, but importantly we observed depletion of the full-length 439 amino acid 64 kDa protein at all three time points post-infection (Fig. 2J). In HCT116 cells, we observed a large depletion of both major forms (as well as a slightly larger likely proteolytic product) of c-Myc at the three time points post-infection (Fig. 2K). In MCF7 cells, the major expressed protein observed was the 64 kDa full-length protein. However, a larger minor protein of approximately 67 kDa was also expressed, consistent with use of an alternative CUG translational start, as has previously been reported [40, 41]. The abundance of both isoforms was dramatically reduced following bacterial-mediated RNAi of c-Myc at all three time-points (Fig. 2L). In summary, SL7207 remained viable and capable of in vitro intracellular colonisation following the chromosomal addition of a shRNA delivery cassette and capable of modifying gene expression in vitro.

**Fig. 3:**
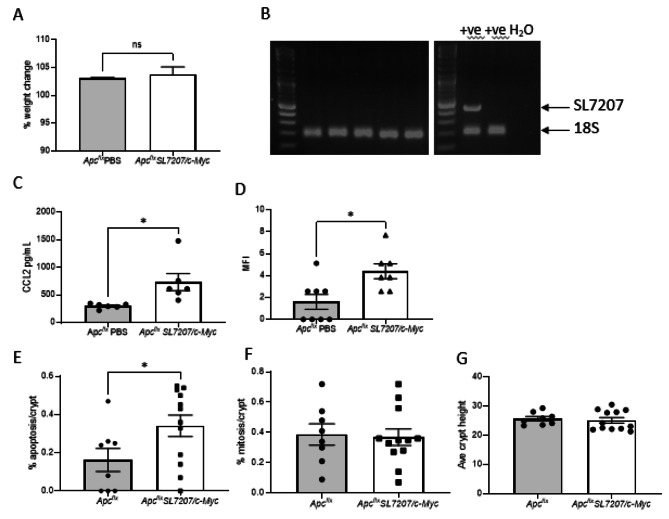
Modified SL7207 strains are well tolerated in vivo. Analysis of mice 7 days after IP administration of PBS vehicle (*Apc*^*+/+*^PBS) or 1 × 10^6^ CFU *SL7207/c-Myc.* (**A**) Weight change data indicating no significant impact on weight gain of *SL7207/c-Myc* (*N* > 3). (**B**) PCR analysis of faecal DNA from mice administered *SL7207/c-Myc* demonstrating clearance of bacteria (Left panel: faecal DNA, Right panel: SL7207, bacteria and water controls). ELISA quantification of serum (**C**) and flow cytometry staining intensity of PBMCs (**D**) for Ccl2 indicating a significant increase in the presence of *SL7207/c-Myc* (t-test pairwise comparisons; *P < 0.01). (**E**-**G**) Quantification of cells in the crypt undergoing apoptosis (**E**), mitosis (**F**) and total crypt cell numbers (**G**)

### SL7207/shRNA strains are well tolerated by healthy mice

As the tumour tropism of *S. typhimurium* SL7207 has been previously demonstrated in vivo [42–44], in immune-deficient xenograft mouse strains, it was important to verify the safety and retention of this phenotype in our recombinants using immunocompetent genetically engineered mouse models of cancer. To assess the therapeutic efficacy *SL7207/c-Myc* toward intestinal tumourigenesis we used immunocompetent *Lgr5creER*^*T2*^*Apc*^*flx/flx*^ mice [46], an acute model of tumourigenesis. In these mice, following tamoxifen (TAM) induction, there is variegated *Apc* loss from the Lgr5^+^ intestinal stem cells (ISCs). ISCs are the cells of origin for colorectal cancer, the loss of *Apc* leads to constitutive activation of the Wnt pathway and is the earliest known event in tumour development. Prior to analysis in TAM induced tumour mouse models (*Apc*^*∆ISC*^), we determined the impact of the bacteria on uninduced control mice (*Apc*^*flx*^). Mice were administered 10^6^ CFU of *SL7207/c-Myc* via ip (*N* = 5; 8–10 weeks old) and at 7 days mice displayed no adverse effects with no impact on weight gain or bacterial presence in faeces (Fig. 3A & B). ELISA and flow cytometry analysis of serum and PBMC for Ccl2 (monocyte chemoattractant protein-1) levels, a key cytokine in the murine response to *Salmonella* infection, indicated significant increases suggesting an appropriate immune response [45] (Fig. 3C & D). Within the large intestine quantification of cellular homeostasis indicated a significant increase in apoptosis (Fig. 3E) following *SL7207/c-Myc* administration that was not reflected in the mitotic rate (Fig. 3F & Supplemental Fig. 1A). However, there was no overall impact on the number of cells within the crypts (Fig. 3G) indicating homeostasis was being maintained. Further administration of 10^6^ CFU of *SL7207/c-Myc* bacteria at weekly intervals had no impact on weight gain over an 8-week period indicating repeated exposure is well tolerated (Supplementary Fig. 1B).

### Bacterial-mediated RNAi of ***c-Myc*** leads to prolonged survival in a mouse model of intestinal tumourigenesis

**Fig. 4:**
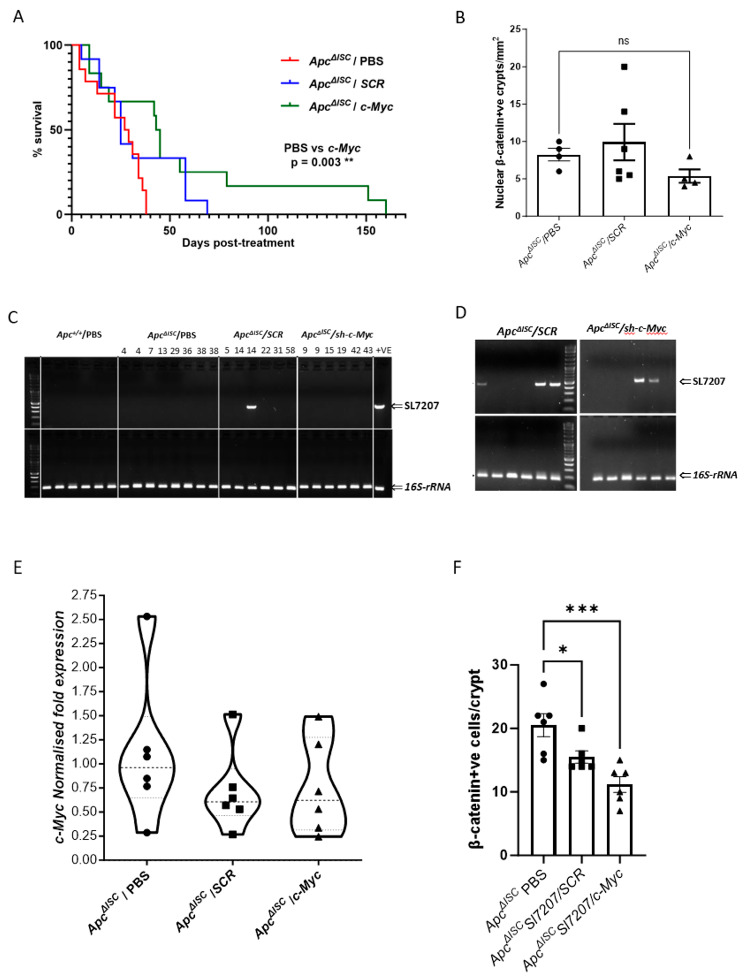
Bacterial-mediated RNAi of *c-Myc* extends survival of *Apc*^*ΔISC*^ mice. (**A**) Kaplan-Meier survival curves for 15-day *Apc*^*ΔISC*^ cohorts administered PBS (*N* = 14, red), 1 × 10^6^ CFU SL7207/SCR (*N* = 12, blue), or 1 × 10^6^ CFU *SL7207/c-Myc* (*N* = 12, green) (Mantel-Cox log rank test (PBS vs. *c-Mycp* = 0.003**, PBS vs. scrambled *p* = 0.197, scrambled vs. *c-Mycp* = 0.319). (**B**) Survival endpoint quantification of crypts from survival analysis staining positive for nuclear β-catenin. (**C**) Survival endpoint PCR analysis of small intestine from survival analysis mice demonstrating general absence of bacteria (survival days indicated). (**D**) PCR DNA analysis of *Apc*^*ΔISC*^ intestine 7 days following bacterial administration indicating variation in bacterial persistence. (**E**) *c-Myc* expression analysis of *Apc*^*ΔISC*^ intestine 7 days after bacterial administration. (**F**) Average number of nuclear β-catenin cells *Apc*^*ΔISC*^ crypts (*N* = 25) 7 days post bacteria administration (two-tailed Mann Whitney U- test, *p < 0.05, ***P < 0.001)

To assess the therapeutic efficacy of the *SL7207/shRNA* strains, bacteria were administered to mice 14 days after induction of ISC *Apc* loss (*Apc*^*∆ISC*^) (Supplemental Fig. 2A), when mice display an extensive “crypt progenitor cell-like” phenotype throughout the intestinal tract [46]. Mice were randomised into three cohorts (*N* > 12), administered either (i) vehicle PBS, or 10^6^ CFU of (ii) *SL7207/SCR*) or (iii) *SL7207/c-Myc*, aged and taken at a defined humane endpoint. Kaplan-Meier survival analysis demonstrated a significant increase in survival between the *SL7207/c-Myc* and control PBS mice with 50% survival rates extending from 30 to 45 days (Log-rank (Mantel-Cox) test *P* = 0.003) (Fig. 4A), with no significant difference between WT v *SL7207/SCR* (*P* = 0.2) and *SL7207/SCR* v *SL7207/c-Myc* (*P* = 0.32). Endpoint analysis indicated that mice succumbed to equivalent tumour burden (Fig. 4B), were negative for bacteria in faeces and had no significant difference in liver or spleen appearance or size (Supplemental Fig. 2B&C). Expression analysis of small intestine at endpoint indicated bacterial persisted only in a single *SL7207/SCR* mouse at day 14 post bacterial administration (Fig. 4C). This suggests that in this model at point of *SL7207* administration the tumour cells either do not contain sufficient levels of aromatic amino acids to overcome the attenuation driven by deletion of *AroA* or the required local immune suppression to support bacterial persistence. Closer inspection of the survival curves highlighted that divergence between PBS and *SL7207/c-Myc* cohorts is not evident until ∼ 25 days post bacteria administration, with ∼ 25% of all mice reaching endpoint within 25 days. This suggests a dichotomy in the cohort, potentially due to induction variation from the *Lgr5creER*^*T2*^ transgene producing a subset of mice in which recombination events cross the threshold that can be impacted by a single *SL7207/c-Myc* administration. To investigate this, we examined mice at 7 days post bacterial administration. At day 7 faecal culture (Supplemental Fig. 2D&E) and expression analysis on 1 cm of proximal small intestine indicated bacterial persistence in 3/6 *SL7207/SCR* and 2/6 *SL7207/c-Myc* treated mice (Fig. 4D). qRT-PCR analysis showed no significant reduction due to *SL7207/c-Myc* shRNA but again closer inspection indicates the presence of two groups, with 3/6 having greater than 2-fold reduction compared to 1/6 for the PBS cohort (Fig. 4E). However, mice administered *SL7207/c-Myc* had significantly less B-catenin nucleated cells compared to *SL7207/SCR* and PBS controls (Fig. 4F). This data is consistent with *SL7207/c-Myc* driven knockdown leading to a transient significant reduction in *Apc* deficient cells (Fig. 4F), as *Apc*^*−/−*^*c-Myc*^*−/−*^ cells are unviable [6], which manifests as an extension of survival. In summary, despite the limitations of this model, our data provides evidence that *SL7207/shRNA* strains can provide a targeted therapeutic benefit effect toward intestinal tumourigenesis, and potentially other solid tumours, without otherwise impacting health.

### Prolonged survival of mice with mammary tumours due to RNAi of cMyc

**Fig. 5:**
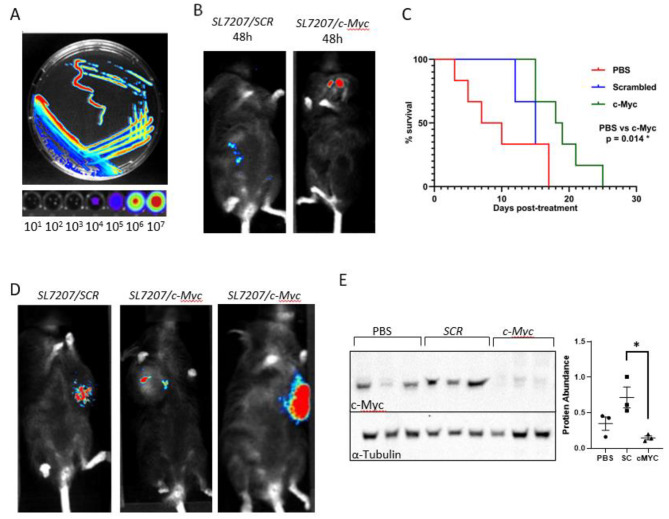
Bacterial-mediated RNAi of *c-Myc* extends survival of *BlgCre BRCA2*^*flx/flx*^*p53*^*flx/flx*^ mice. (**A**) *SL7207/c-Myc(lux)* expressing imaged as surface-grown culture (top panel) and dilutions of a submerged culture (bottom panel; cells/ml indicated. (**B**) In vivo luminescence imaging of tumour bearing *BlgCre BRCA2*^*flx/flx*^*p53*^*flx/flx*^ mice 48 h after ip administration of 10^6^ CFU of *SL7207/c-Myc(lux).* (**C**) Kaplan-meier survival curve indicating a significant increase in survival of mammary tumour mice delivered a single ip dose of 10^6^ CFU of *SL7207/c-Myc(lux)(green)* versus control *SL7207/SCR(lux*) (blue; *P* = 0.014) and PBS (red; *P* = 0.023) mice (PBS vs. Scrambled *P* = 0.37; Mantel-Cox log rank test). (**D**) Luminescent imaging demonstrating persistence *SL7207* bacteria in mammary tumours > 14 days following ip administration. (**E**) Western immunoblots (left panel) and densitometry quantification (right panel) of homogenised tumours indicating significant loss of c-Myc protein in *SL7207/c-Myc(lux)* colonised mammary tumours (*P* = 0.0175)

To demonstrate the potential of our *SL7207/shRNA* strains for treatment of other solid tumours we administered bacteria to a small cohort of immunocompetent *BlgCreBrca2*^*flx/flx*^*p53*^*flx/flx*^ mice, an autochthonous model of mammary tumourigenesis [47]. To enable non-invasive monitoring of localisation of the bacteria and demonstrate tumour tropism, a synthetic bacterial luciferase operon expressed under control of a strong constitutive promoter was integrated at the chromosomal 16 S rRNA locus (*SL7207/(lux)*) [48]. Bacterial bioluminescence was verified with both surface-grown and serial dilutions of submerged cultures (Fig. 5A). To demonstrate tumour tropism, mice with palpable mammary tumours within 10% of the approved humane endpoint threshold were administered PBS or 10^6^ CFU of *SL7207/SCR(lux)* or *SL7207/c-Myc(lux)* and imaged using the PhotonIMAGER Optima system. At 48 h bacteria had specifically colonised mammary tumours demonstrating the ability of the bacteria to migrate from the ip injection point (Fig. 5B & Supplemental Fig. 3A-D). Following confirmation of the bacteria’s mammary tumour tropism further cohorts (N = > 4) of mice were treated and aged until the tumour attained the specified humane endpoint threshold (Fig. 5C). Akin to the intestinal tumourigenesis data the cohort administered *SL7207/c-Myc* had significantly extended survival compared to mice treated with PBS (*P* = 0.014) and *SL7207/SCR* (*P* = 0.023); with no difference between WT v *SL7207/SCR* (*P* = 0.371). In this model in vivo imaging indicated persistence of bacteria within the tumour at the endpoint (Fig. 5D). With subsequent resection and western blot analysis of tumours confirmed reduced levels of c-Myc protein in tumours colonised with *SL7207/c-Myc* (Fig. 5E). In summary these data indicate the ability of bacteria to specifically colonise tumours distant from the bacterial entry point and therapeutically knockdown target genes via RNAi.

## Discussion

The attenuated SL7207 strain of *S. typhimurium* was originally developed as a live vaccine [49, 50] and for delivery of DNA vaccines [51]. It is an auxotrophic mutant resulting from deletion of *aroA* and consequently has a growth requirement for two compounds: ρ-aminobenzoic acid and 2,3-dihydroxybenzoate, which the bacteria cannot acquire in sufficient amounts to enable growth in healthy tissue. But, if sufficient bacteria are administered, they can deliver a transient DNA payload to these healthy cells. Due to the deregulated metabolism of tumour cells, these nutrients are available and can complement the metabolic deficiency of the bacteria when they colonise this niche, thereby supporting bacterial growth. Consequently, several studies have identified the tumour-preferential localization of SL7207 when administered intravenously [23, 42–44, 52–55], although in each case using athymic xenograft mouse models. Here we demonstrate that SL7207 derivatives in which a synthetic shRNA expression cassette is stably integrated at the bacterial *xylA* locus also exhibit exquisite tumour-tropism in immunocompetent mouse models of colorectal and breast cancer. The use of immunocompetent mouse models is an important step towards eventual clinical translation of bacterial immunotherapy as it demonstrates that bacteria administered systemically are not destroyed by an animal’s immune system prior to them colonising a tumour. Moreover, subsequent tumour regression is believed to be in part due to immune stimulation by the colonising bacteria [24–26].

In addition, these bacterial strains are competent for cell invasion as demonstrated by their efficient colonisation of three tumour cell lines grown in vitro. These in vitro studies revealed significant RNAi of *c-Myc* indicating that enough shRNA is synthesised and released by the bacteria growing intracellularly. In addition, in the SW480 colorectal cell line, bacterial mediated RNAi of *c-Myc* resulted in the appearance of a proteolytic derivative c-Myc-nick protein. In colon cancer, the appearance of Myc-nick is known to be enhanced under stress conditions such as hypoxia and nutrient deprivation [39]. It is likely that a combination of bacterial colonisation and depletion of c-Myc in SW480 cells is a stress condition that promotes this proteolysis. It is of note here that we have used only a single shRNA sequence construct to provide a proof of principle. Further research may identify sequences or combination of sequences with even higher RNAi efficiency. With the specificity of delivery to tumour tissue avoiding the liver toxicity that has been previously reported after systemic delivery of shRNA [56, 57]. Perhaps even more compelling evidence for bacterial mediated RNAi comes from analysis of c-Myc protein abundance in mammary tumours colonised by the bacteria. In this in vivo experiment, we detected very little protein due to knockdown of *c-Myc* expression.

Chromosomal integration of a shRNA expression cassette is intended to provide prolonged RNAi of a target gene in cells colonised by the bacteria. Evidence for the efficacy of targeting c-Myc expression was demonstrated by the prolonged survival of mice with either mammary or colorectal tumours after bacterial treatment. Treatment with bacteria producing scrambled shRNA resulted in a moderate increase in survival, presumably because of bacterial immunotherapy. However, the mouse cohorts treated with bacteria expressing shRNA targeting *c-Myc* had greatly extended survival times compared to the scrambled shRNA treatment cohort, with variance attributable to large differences in tumour burden between individual mice at the time of treatment. Bacterial treatment evidently slowed tumour growth, extending survival until the maximum tumour size allowed as a humane endpoint was reached. Moreover, we observed that the bacteria can affect very early stages of intestinal tumorigenesis by depleting numbers of β-catenin-positive ISCs, suggesting a possible prophylactic role for the treatment. This is a positive finding when you consider the limitations of the *Lgr5cre* driven acute model of intestinal tumourigenesis which displays variegation in recombination, lacks discrete tumours and a relevant tumour microenvironment. While the ability of a single administration of *SL7207/c-Myc* to make an impact in this setting is noteworthy it is acknowledged that this model, in contrast to the mammary data, did not provide in vivo confirmation of efficient intestinal epithelial RNAi. To overcome this limitation future experiments should consider earlier timepoints, multiple dosing or the use of more physiological relevant intestinal tumour models. Such as *Apc* loss driven by *Car1cre*, which produces single orthotopic large intestine polyps [58], akin to human presentation at clinic. However, it is of note we demonstrate the ability to therapeutically target c-Myc, which regulates the expression of the β-catenin co-transcriptional factor LEF1, required for the retention of β-catenin in the nucleus, thereby potentiating activation of the WNT pathway that drives CRC [59]. The c-Myc transcription factor directly or indirectly regulates thousands of genes that influence proliferative signalling, metabolism, angiogenesis, invasion and survival [3, 60, 61] and hence is critical for maintenance of the neoplastic state, giving rise to the concept of ‘oncogene addiction’ [62]. c-Myc addiction has been studied in experimental transgenic mouse models whereby the oncogene can be conditionally expressed, using the Tet System, demonstrating that inactivation of this oncogene leads to tumour regression [5]. However, c-Myc inactivation in genetically engineered conditional mouse hematopoietic tumours failed to induce sustained tumour regression in the absence of an intact host immune system [63]. This can be rationalised in the knowledge that c-Myc has a key tumour-specific role in regulating immune suppression by upregulating immune checkpoint proteins PD-L1 and CD47 [64]. Therefore, RNAi of c-Myc can not only impact intrinsic characteristics of tumour cell biology, but also stimulate the host immune system to attack the tumour; as a consequence, bacterial delivery of this RNAi can be considered as an enhanced bacterial immunotherapy. In support of this position is our mammary data which shows a trend for increased survival and *c-Myc* expression in tumours infected with *SL7207/SCR*. Macrophages are associated with poor prognosis in breast cancer and contribute to shaping the anti-tumour response [65]. *Salmonella* leads to upregulation of *c-Myc* in macrophages and their cell death [66], data consistent with our survival curve which shows a trend for extended survival (Fig. 5C). However, further research is required to confirm this and establish whether it is an effective angle for this therapeutic approach.

While we report RNAi of just a single oncogene in tumours, this enhanced bacterial cancer therapy offers a platform for targeting many other oncogenes and combinations thereof, as the bacterial payload can be modified accordingly. As a consequence, it provides both a means to detect tumours by virtue of their colonisation by reporter bacteria, but also a tangible approach towards eventual personalised precision therapy guided by assessment of patient-specific tumour biomarkers. Further work prior to progression to the clinic is now required to demonstrate safety, understand the immune consequences, and further understand the mechanism that underpins the tumour tropism and colonisation.

## Methods

### Derivation of recombinant ***Salmonella typhimurium*** SL7207 strains

*Salmonella typhimurium* SL7207 was transformed with the plasmid p16s*lux* [48] at 30^o^C, the permissive temperature for plasmid replication. An erythromycin-resistant colony was incubated in LB broth at the non-permissive 42^o^C and erythromycin-resistant colonies (SL7207 Lux^+^) obtained in which the plasmid had integrated at the 16S locus of the chromosome. SL7207 Lux^+^ was transformed with plasmid pKD46 [37]. This plasmid was used to facilitate arabinose-inducible lambda red recombinase-dependent disruption of the chromosomal *xylA* gene with PCR products containing short flanking homology with the *xylA* locus. Encoded within the synthesised expression cassettes are a *tac* promoter upstream of scrambled shRNA (TGTGGCGGCGCATAAGAAGCATATTTCAAGAGAATATGCTTCTTATGCGCCGTTTTTGGTGGT) or *cMyc* shRNA sequences (cMyc_Hs:TGTGGAATTGTGATGTCAAGAGGCGAACACACTTCAAGAGAGTGTGTTCGCCTCTTGACATTTTTTGGTGGT or cMyc_Hs_Mm:TGTGGAAGAGCAAGAAGATGAGGATTCAAGAGACTTCCTCATCTTCTTGCTCTTTTTGGTGGT and a kanamycin resistance gene. The linear DNA cassette was amplified by PCR using Q5 DNA polymerase from NEB (#M0491) with primers that have overhanging 5’ and 3’ ends encoding homology arms upstream and downstream of the SL7207 x*ylA* locus (Supplementary Table 1). These PCR products were gel purified, then electroporated into SL7207 Lux^+^; pKD46 that had previously been induced with arabinose. The pKD46 plasmid, that has a temperature sensitive origin of replication, was subsequently cured from kanamycin resistant clones by culturing at 37 °C in super optimal broth with catabolite repression (SOC). Resultant colonies were tested by colony PCR, using both internal primers and integration primers (Supplementary Table 1).

To compare stability of a cassette integrated at the *xylA* locus with a plasmid, strains containing the scrambled shRNA cassette and another containing plasmid pME6 [38] with the same kanamycin resistance gene were cultured in triplicate in non-selective LB broth, and then sub-cultured every 24 h. After 10 days, the proportion of kanamycin resistant cells in each culture was enumerated by plating dilution series on LB with and without kanamycin. All oligo sequences available upon request.

The GFP-expressing strain used to monitor cell invasion was created by introduction of a multi-copy plasmid, pdagGFP [67] encoding the fluorescent protein.

### Tissue culture

SW480 cells [American Type Culture Collection (ATCC) #CCL-228], HCT116 (ATCC-CCL-247) and MCF7 (ATCC -HTB-22) were cultured in Dulbecco’s modified Eagle’s Medium (DMEM; Life Technologies) supplemented with 10% Foetal Bovine Serum (FBS, LifeTechnologies) and PenStrep. All cell lines were cultured at 37 °C with 5% CO2 in a humidified incubator.

### Co-culture of SL7207/GFP with SW480, HCT116 and MCF7 cell lines

Firstly, bacteria grown in LB broth were enumerated at several time points of culture. Briefly, 0.1 ml of three dilutions of 10^− 5^, 10^− 6^ and 10^− 7^ for each timepoint were spread on LB kanamycin 50 μg/ml agar plates to achieve single colonies. Plates of the highest dilution fell within the 30–300 colonies per plate statistically valid range. The number of bacteria for each OD_600_/timepoint was calculated by multiplying by the dilution factor; supplemental method Fig. 1A shows the number of bacteria versus OD_600_. Once bacteria had been enumerated, the multiplicities of infection (MOI) that would achieve the highest number of bacteria per mammalian cell were optimised. To establish the MOI, cultured cells were seeded into 10 ml DMEM PenStrep in T75 flasks 48 h before co-culture, and on the day of co-culture viable cells were counted using a haemocytometer and trypan blue. SL7207 strains were enumerated measuring their optical density at 600 nm and calculating the number of bacterial cells present at this OD. The number of bacterial cells required to achieve MOIs of 1000:1, 100:1 and 10:1 was calculated and bacteria were incubated with cells for 0.5, 1 and 2 h before extra-cellular bacteria were removed, followed by several PBS washes and incubation with DMEM (supplemented with gentamicin (200 μg/ml) to kill any remaining extracellular or attached bacteria). To determine the number of bacteria per cell, cells were lysed in PBS containing 1% Triton X100 and the dilutions of the lysates were plated on LB agar containing kanamycin (50 μg/ml) with colony numbers enumerated the following day. Supplementary Fig. 3B shows that an MOI of 1,000 bacterial cells to 1 mammalian cell was optimal for achieving the highest bacterial to mammalian cell ratio.

*S. typhimurium* SL7207 strains expressing scrambled-shRNA and cMyc-shRNA were revived from glycerol stocks 12 h before scheduled co-culture. A scraping of a glycerol stock was placed in 5 ml of LB broth with kanamycin (50 μg/ml) and grown in a shaking incubator at 37 °C for 8 h. 3 ml of the 8-hour culture was diluted into 300 ml of LB and kanamycin and left to grow in a shaking incubator at 37 °C until the following morning. At this point the MOI was calculated as outlined above. The SL7207 strains were centrifuged at low speed (900xg) for 10 min and resuspended at the required MOI in DMEM cell culture medium with kanamycin (50 μg/ml) in place of PenStrep. The seeding culture medium was removed and replaced with 10 ml DMEM kanamycin (50 μg/ml) containing the respective SL7207 strains. These were incubated at 37 °C 5% CO2 for two hours. After two hours, the media was removed and replaced with DMEM supplemented with gentamicin (200 μg/ml) and incubated for 1 h to kill extracellular and attached bacteria. The gentamicin DMEM was then removed and replaced with kanamycin DMEM. Timepoint 0 was when the gentamicin DMEM was removed. RNA and protein harvests were performed subsequently at 24 h, 48 and 72 h. Experiments were repeated in triplicate for each time-point.

### RNA extraction and quantitative real-time PCR analysis

Total RNA was extracted using a RNeasy Plus Mini Kit (Qiagen) following the manufacturer’s instructions. Briefly, cells were washed with PBS in a 6-well plate. To avoid any gene expression changes during trypsinisation, cells were lysed directly with the highly denaturing RLT (RNase Later) plus buffer which simultaneously inactivates RNases. The volume of buffer was adjusted depending on cell number (350 μl for 5 × 10^6^ cells; 600 μl for up to 1 × 10^7^ cells).

Lysates were passed through a gDNA eliminator spin column to eliminate genomic DNA. The flow-through was then applied to a RNeasy spin column after the addition of an equal volume of 70% ethanol followed by two wash steps before elution. The final RNA purity (260/280nm ratio of ∼ 2) and concentration was determined using a NanoDrop 2000c. First strand cDNA synthesis from an RNA template was carried out using the iScript™ cDNA Synthesis Kit from BioRad. 1 μg of RNA was reverse transcribed using oligo(dT) primers, thus excluding amplifying prokaryotic transcripts in the RNA extract. Samples were treated with RNase H to eliminate residual RNA. The final cDNA was then diluted 1 in 8 with nuclease-free water. Non-reverse transcriptase samples were included for all samples with no amplification being observed in subsequent qPCR (quantitative real-time polymerase chain reactions). qPCR reactions were performed using either iQ SYBR Green Supermix (BioRad) in a CFX96 Real-Time PCR Detection System C100 (Biorad) or GoTaq qPCR Master Mix (Promega) in the QuantStudio™ 7 Flex Real-Time PCR System (Applied Biosystems). Triplicate reactions were prepared for each sample in a final volume of 20 μl. Each reaction consisted of 1x qPCR Master mix, 1.5 μl of diluted cDNA (equivalent to 9.375ng/μl of RNA), and primers to a final concentration of 0.1μM. The PCR program consisted of an initial denaturation at 95 °C for 10 min, followed by 40 cycles of 95 °C for 15 s and 60 °C for 1 min. This was followed by melt curve analysis from 60 to 95 °C (in 0.5 °C increments). Custom-designed primers and commercial primers (Quantitect Primer Assays, Qiagen) were used in this study with primers used listed in Supplementary Table 1. Differences between groups were assessed using the 2 − ΔΔCT method [68]. Two-tailed Mann–Whitney U (MW) tests were performed on the ΔCt values and differences with *P* values less than 0.05 were considered significant [69].

### Protein extraction and western blotting

M-PER lysis buffer (ThermoFisher Scientific) was used to prepare whole-cell lysates, a proprietary non-denaturing detergent in 25nM bicine buffer (pH 7.6). To counter protein degradation and dephosphorylation the buffer was supplemented with protease and phosphatase inhibitors (Halt Protease Inhibitor Cocktail and Phosphatase Inhibitor Cocktail, ThermoFisher Scientific) following the.

manufacturer’s protocol. For Tissue Western Blots, a 100-200 mg piece of tumour tissue was manually homogenised using scalpels into 1-2 mm pieces. This was transferred to Lysing Matrix D tube (MP Bio) RIPA buffer. Samples were further homogenised using FastPrep-24 for 3 × 40s at a speed setting of 6 m/s. Tubes were cooled on ice between runs. Homogenates were centrifuged at 14,000xg for 10 min and supernatants collected. A BCA (bicinchonionic acid) assay (BCA protein assay kit, ThermoFisher Scientific) was used to measure protein concentrations of whole cell lysates and supernatants from tissue homogenates using a nanodrop spectrophotometer. For each sample 30 μg of protein was used. These samples were made up to 13 μl with water with 2 μl reducing agent and (Bolt Sample Reducing Agent, ThermoFisher Scientific) and 5 μl Sample Buffer (Bolt LDS Sample Buffer, ThermoFisher Scientific). The samples were heated to 70 °C for 10 min before electrophoresis. A precision plus protein dual colour standards (Biorad) protein ladder was run alongside the samples in a 4–12% precast polyacrylamide gel (ThermoFisher Scientific) immersed in 2-(N-morpholino) ethanesulfonic acid (MES DSD Running Buffer, ThermoFisher Scientific) for.

30 min at 100 V. Proteins were then transferred to a PVDF (Polyvinylidene fluoride) membrane (Immobilon-P, Millipore) using a wet transfer system (Biorad) at 1 A for 90 min in transfer buffer (25mM Tris, 192mM Glycine). PVDF membranes were soaked in 100% methanol prior to transfer. Membranes were blocked for one hour at room temperature with Milk Solution (10% dry milk in PBS/ 0.5% Tween 20). Primary antibodies were diluted in milk solution or 0.5% Tween 20 with 5% Bovine Serum Albumin (BSA) and incubated with the membrane overnight at 4 °C. The membrane was blocked for 10 min in milk solution before adding HRP (Horse Radish Peroxidase)-conjugated secondary antibody that was also diluted in milk solution and incubated for one hour at room temperature. Finally, three 10 min washes were performed using Tween20 PBS (Phosphate-buffered saline) solution (0.1% Tween in 1x PBS) to remove any non-specifically bound and unbound antibodies. A list detailing all the antibodies used for Western Blotting is shown in Supplementary Table 2. Western Lightning Plus-ECL (Perkin Elmer) was used as a substrate for the HRP-conjugated secondary antibodies following the manufacturer’s protocol. Chemiluminescent signals were detected using digital camera detection with a Biorad ChemiDoc XRS + imaging system following densitometry analysis data was compared using Tukey’s multiple comparisons test.

### Immunofluorescence imaging

SW480, HCT116 and MCF7 cells were cultured in Ibidi cells in focus 24 well μ-plate (#82,406). Cells were untreated or treated with SL7207 containing a pUC18 GFP plasmid, at an MOI of 1000:1. After 2 h of co-culture, extracellular bacteria were killed with the addition of gentamycin 200 mg/ml for an hour. Cells were fixed with 4% PFA and stained with α-tubulin (Cell Signalling 3873, 1:2000 and Cell Signalling 4409 1:1000) followed by DAPI Prolong gold antifade (Invitrogen P36931).

In Lab-Tek II chamber slides (ThermoFisher 154,526), SW480, HCT116 and MCF7 cells were left untreated, or treated with *SL7207/SCR* or *SL7207/cMyc* strains at an MOI of 1000:1. After 2 h of co-culture, extracellular bacteria were killed with the addition of gentamicin 200 mg/ml for an hour. At 24, 48 and 72 h post co-culture, cells were fixed with 4% PFA, and stained with α-tubulin (Cell Signalling 3873, 1:3000 and Cell Signalling 4413 1:1000) and cMyc (Cell Signalling 5605, 1:1000 and Cell Signalling 4408 1:1000) antibodies followed by DAPI Prolong gold antifade (Invitrogen P36931). All confocal images were acquired using Zeiss LSM 880 Airyscan.

### In vivo studies

All animal procedures were conducted in accordance with institutional animal care and reported in accordance with NC3R(UK) ARRIVE guidelines. Work was approved by a UK Home Office Project license (PBEB09FBB; protocol 3). Animals were maintained on an outbred background and housed in a standard facility under a 12 h light cycle, with water and chow *ad libitum*. Where applicable both male and female mice were used for this study. Two types of animal models were used in the study and based on a sample size power analysis (Tail(s) = Two; Effect size |ρ|=0.9; α err prob = 0.05; Power (1-β err prob) = 0.95) the treatment groups consisted of a minimum of 6 mice. For both models, 10^6^ CFU bacteria suspended in 100 μl PBS were administered by intraperitoneal injection. No fatalities because of this administration were observed. Mice were weighed both before and 48 h after treatment with no significant weight changes observed. For intestinal tumourigenesis the tamoxifen inducible *Lgr5creER*^*T2*^*Apc*^*flx/flx*^ was used [70, 71]. Conditional knockout of *Apc* in crypt progenitor cells is induced after intra-peritoneal (ip) injection with 10 mg/kg of tamoxifen. With a “crypt progenitor” phenotype rapidly formed along the whole length of the intestine after deletion of *Apc*. Induced mice were selected randomly for each treatment group. Mice were then injected ip with respective SL7207 strains 14 days post induction for analysis. Mice were harvested at a humane endpoint when mice displayed phenotypes indicative of tumour burden (pale feet, bloating, prolonged weight loss, prolapse or piloerection).

The breast cancer model (conditional *Brca2/p53* knockout under control of *Blg-cre* transgene) develops autochthonous tumours on any of the 5 pairs of mammary glands between 6 and 15 months of age. Palpable tumours were measured [47] and mice with maximum tumour widths or lengths of between 8 and 11 mm were selected for treatments. The humane endpoint at which these mice were culled was when the tumours reached 16 mm in width or length. PCR conditions for genotyping of the *Blg-cre* transgene and the conditional alleles for *Brca2* and *p53* have already been described [47]. Mice were imaged on the photon imager optima (BioSpace Lab, FRA).

### Blood analysis

For flow cytometry peripheral blood was collected from healthy mice 7 days post-administration of either SL7207 or PBS. Peripheral blood mononuclear cells (PBMCs) were isolated with Histopaque®-1119 by density gradient centrifugation within 2 h of sample collection. Isolated PBMCs were stained with 7-AAD and APC-conjugated anti-CCL2 antibody (Miltenyi Biotec, UK; #130-107-483, clone REA485). Samples were analysed using a BD LSRFortessa™ Cell Analyzer. For Ccl2 serum analysis we used the MCP-1/CCL2 Mouse Uncoated ELISA Kit according to manufacturer instructions (Thermo-Fisher, UK; #88-7391) and plates visualised with the Clariostar plate reader m(BMG, UK).

### Intestinal cell analysis

For immunohistochemistry (IHC), tissue was fixed in 10% neutral buffered formalin (Sigma, UK) and processed by conventional means. The following antibodies were used to stain for: *Apc* deficient cells anti-β-catenin (Transduction Lab #610,154) and proliferation anti-Ki67 (Abcam #ab16667) Protocols were available upon request. Cellular analysis was performed within small intestinal tumours or on a total of 25 crypts from the first 5 cm of small intestine. Cellular analysis was performed on > 25 whole crypts from at least three mice of each genotype. Apoptotic and mitotic index were scored from haematoxylin-and-eosin (H&E)-stained sections as previously described [72].

### Electronic supplementary material

Below is the link to the electronic supplementary material.

Supplementary Material 1

## Data Availability

The datasets generated during and/or analysed during the current study are all fully disclosed within manuscript. Requests for data and materials will be considered taken into consideration the relevant patent and IP.

## Abbreviations

CFU: Colony forming units
CRC: Colorectal cancer
ip: Intraperitoneal injection
RNAi: RNA interference
shRNA: Short hairpin RNA
