# Supplementary material for: Enhanced bacterial cancer therapy delivering therapeutic RNA interference of c-Myc
Source: Cell Biosci. 2024 Mar 23;14:38. doi: 10.1186/s13578-024-01206-8 (PMC10961001; doi:10.1186/s13578-024-01206-8)
Supplement: Supplementary file 1 — Supplementary Material 1 [file 13578_2024_1206_MOESM1_ESM.docx]

**
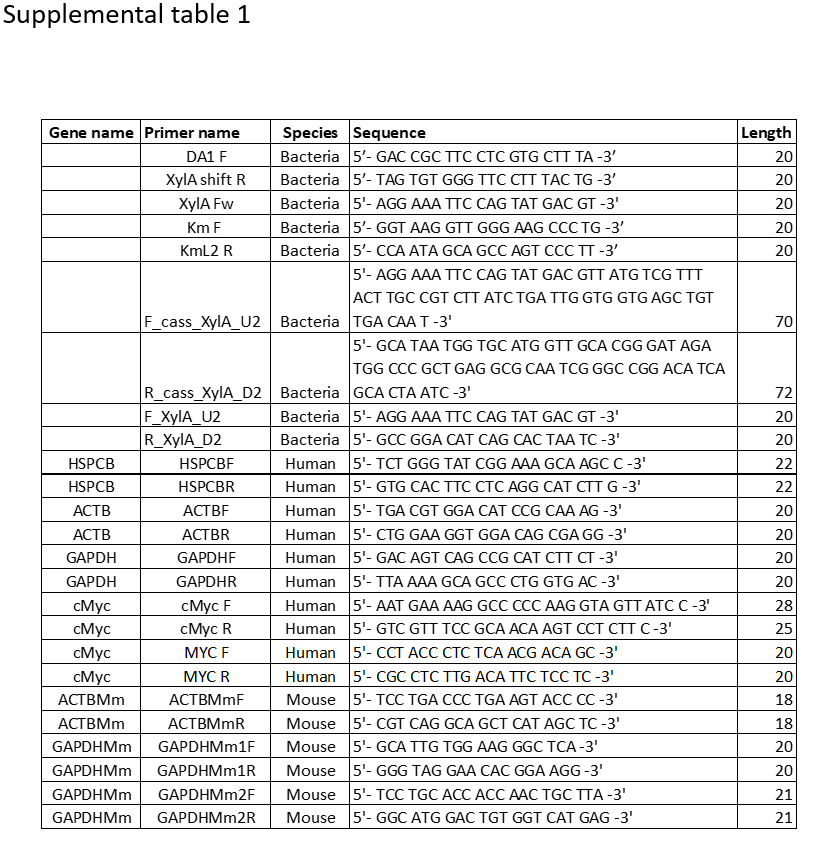
Supplemental Table 1: List of primers**. Bacterial primers were used to verify integration into the bacterial chromosome. Human and mouse-specific primers were used for qRT-PCR.

**
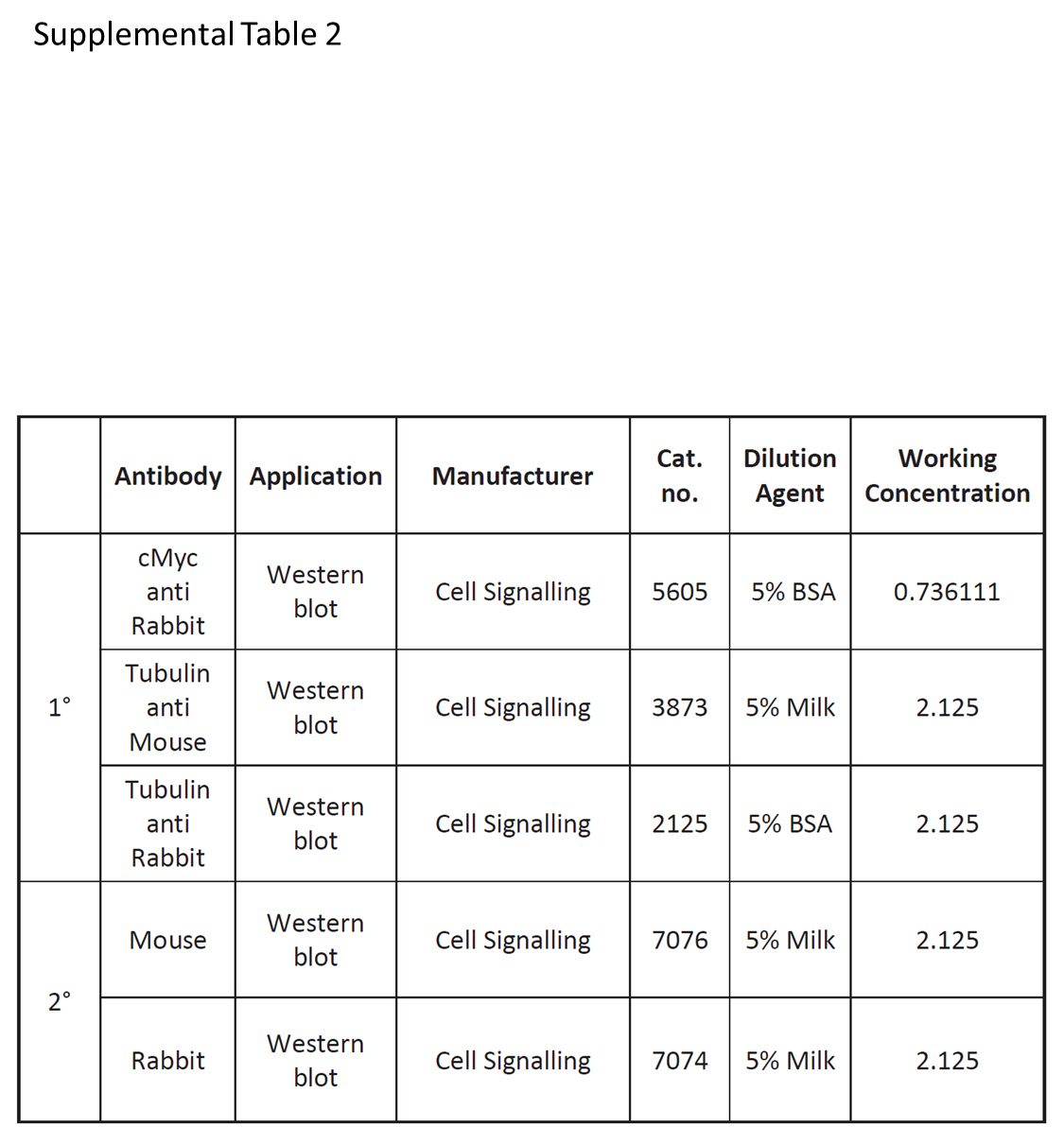
**
**Supplemental Table 2.** List of antibodies used for western blot analysis with details of species raised in and catalogue numbers.

Supplemental Figure 1


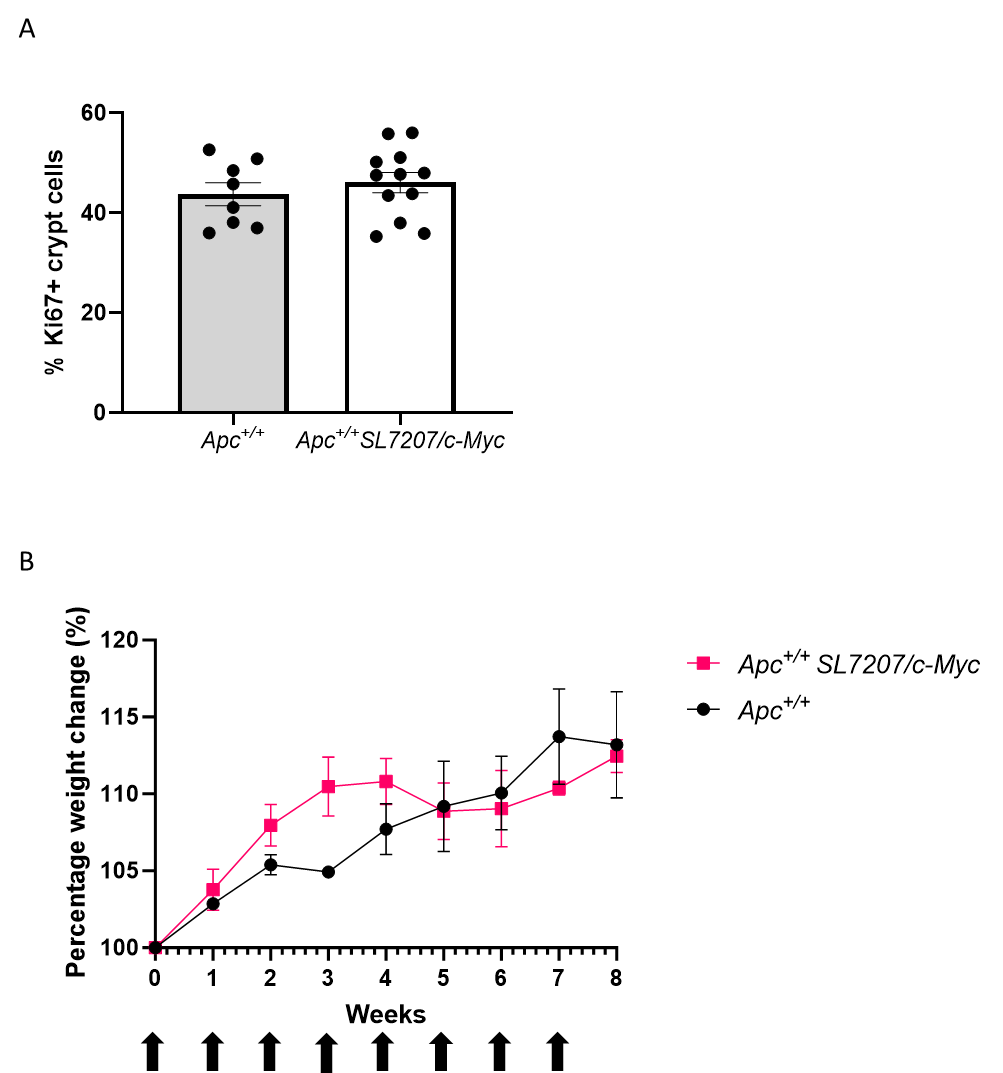


**Supplemental Figure 1.** (A) Quantification of Ki67+ve cells in small intestine crypts 7 days post administration of bacteria. (B) Weight change over 8-week period of wild type *Apc^+/+^* mice receiving weekly administrations of 10^6^ CFU of *SL7207/c-Myc* via ip (arrow indicate times of administrations).

**
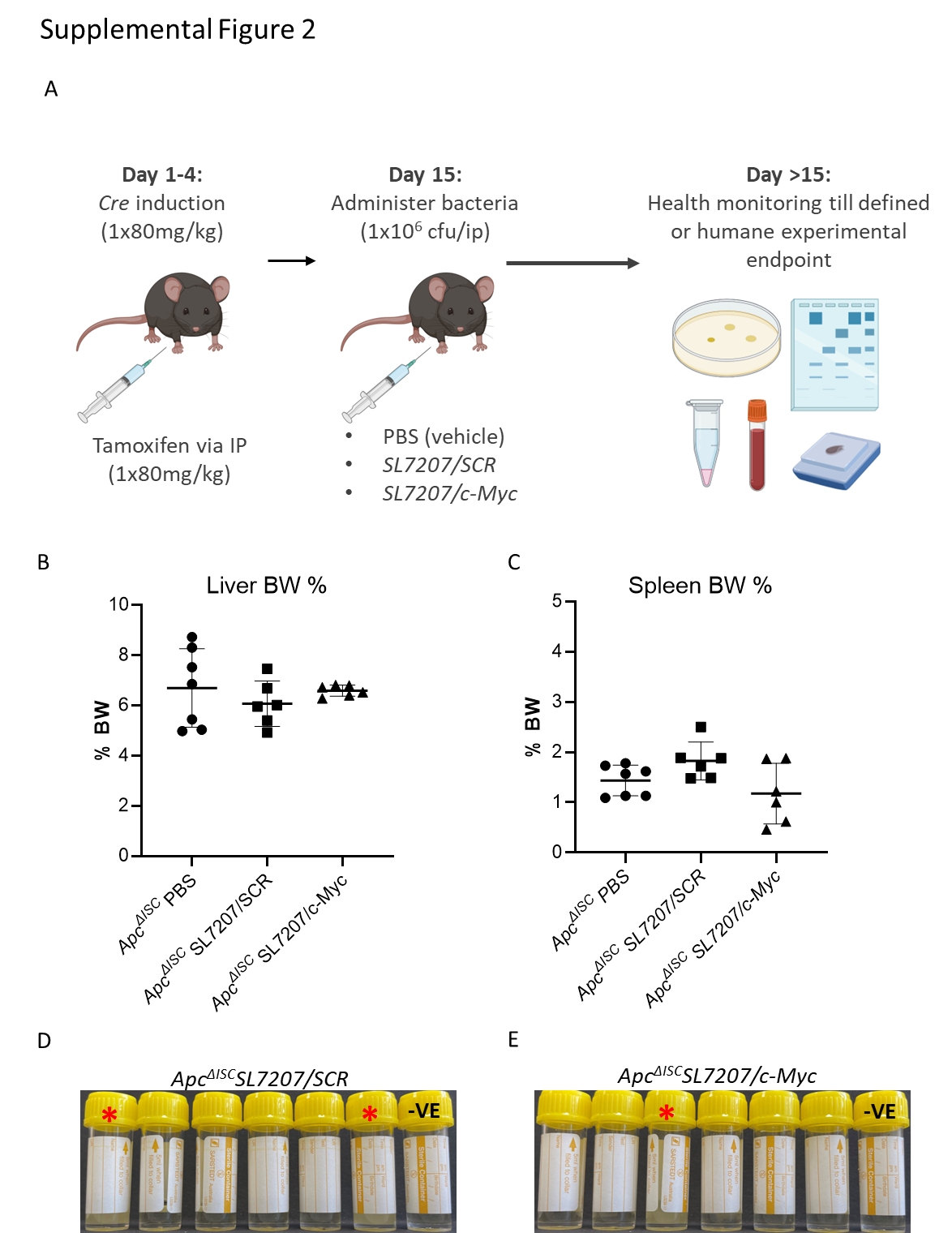
**

**Supplemental Figure 2.** (A) Schematic showing *Cre* induction and bacterial administration regimes for *in vivo* experiments. Endpoint liver (B) and spleen (C) morphometrics from *Apc^∆ISC^* survival experiment indicate no impact of a single administration of recombinant SL7207 strains. SL7207 selective faecal cultures of *Apc^∆ISC^* mice 7 days after bacterial administration of (D) *SL7207/SCR* and (E) *SL7207/c-Myc* (* denotes positive culture).

**
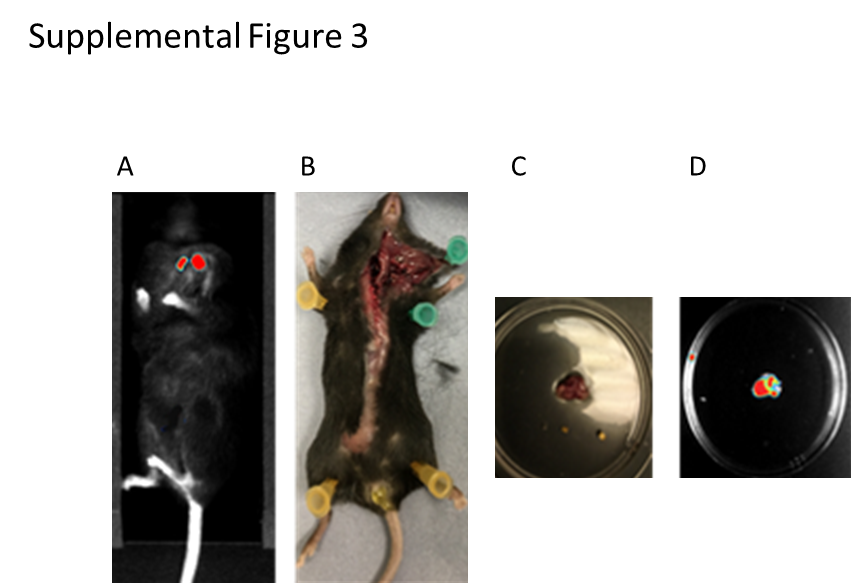
**
**Supplemental Figure 3**. 1x10^6^ cells of *SL7207/c-Myc(lux)* were administered ip to an induced *BlgCre Brca2^flx/flx^ p53^flx/flx^* tumour bearing mouse and monitored via brightfield and luminescent imaging. An *in vivo* image of representative mouse 48 h after bacterial administration (A), that was sacrificed to resect the tumour (B&C) and was subsequently re-imaged (C&D).

**
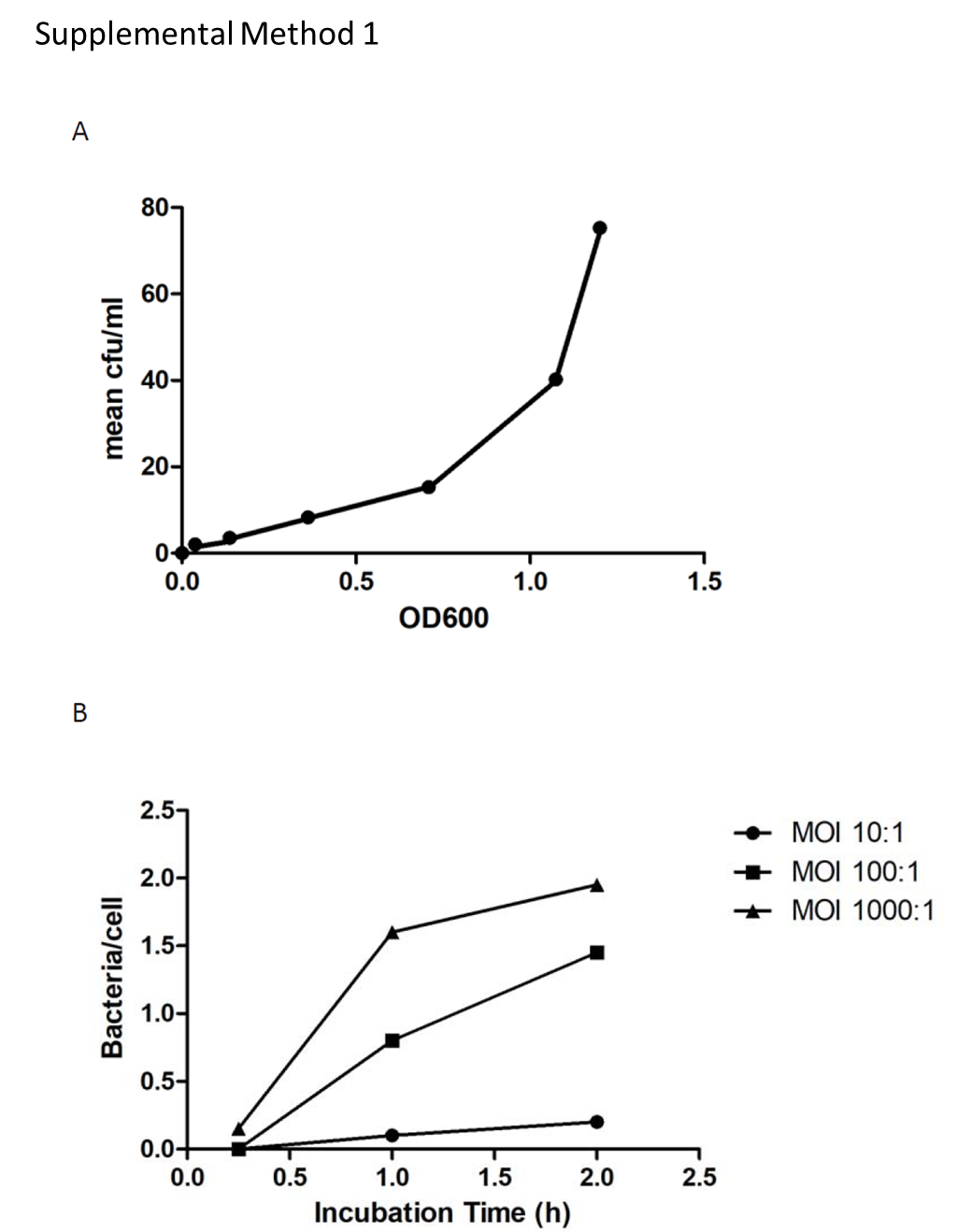
**

**Supplemental Method 1**. Optimization of bacterial co-culture conditions. (A) Graph plotting bacterial optical density against numbers of colony forming units (CFU). (B) Graph plotting numbers of bacteria per cell for different multiplicities of infection (MOI) for different incubation times.
